# Statement of the Italian Association of Medical Physics (AIFM) task group on radiation dose monitoring systems

**DOI:** 10.1186/s13244-022-01155-1

**Published:** 2022-02-05

**Authors:** Francesco Ria, Loredana D’Ercole, Daniela Origgi, Nicoletta Paruccini, Luisa Pierotti, Osvaldo Rampado, Veronica Rossetti, Sabina Strocchi, Alberto Torresin, Association of Medical Physics Task Group, Alberto Torresin, Luisa Pierotti, Giacomo Belli, Paola Bregant, Paola Isoardi, Alberto Mari, Andrea Nitrosi, Luca Nocetti, Nicoletta Paruccini, Maria Grazia Quattrocchi, Anna Radice, Osvaldo Rampado, Nicoletta Scrittori, Chiara Sottocornola, Sabina Strocchi, Marina Sutto, Giovanna Zatelli, Domenico Acchiappati, Rosa Antonella Aoja, Marco Brambilla, Marco Branchini, Vittorio Cannatà, Tiziana Costi, Claudia Cutaia, Loredana D.’Ercole, Antonella Del Vecchio, Stefania Delle Canne, Maria Di Pasquale, Silvia Elisabetta, Cinzia Fabbri, Maria Daniela Faico, Daniele Fantinato, Caterina Ghetti, Marco Giannelli, Carlo Giordano, Simone Grisotto, Gabriele Guidi, Francesco Lisciandro, Luigi Manco, Pier Giorgio Marini, Paola Moresco, Nadia Oberhofer, Daniela Origgi, Francesca Palleri, Claudia Pasquali, Massimo Pasquino, Andrea Peruzzo, Giuseppe Petrillo, Silvia Pini, Daniela Rembado, Francesco Ria, Lucia Riccardi, Raffaella Rosasco, Giulio Serelli, Raffaella Soavi, Michele Stasi, Adriana Taddeucci, Eugenia Tonini, Annalisa Trianni, Piera Turano, Giovanna Venturi, Daniele Zefiro, Felicia Zito

**Affiliations:** 1 Carl E. Ravin Advanced Imaging Labs and Clinical Imaging Physics Group, Duke University Health System, 2424 Erwin Road, Suite 302, Durham, NC 27710 USA; 2 UOC Fisica Sanitaria, Fondazione IRCCS Policlinico San Matteo, Viale Golgi 19, 27100 Pavia, Italy; 3 Medical Physics Unit, European Institute of Oncology (IEO), IRCCS, Via Ripamonti 435, 20141 Milan, Italy; 4 S.C. Fisica Sanitaria, ASST Monza, via Pergolesi 33, 20900 Monza, Italy; 5 Department of Medical Physics, IRCCS Azienda Ospedaliero-Universitaria di Bologna, S.Orsola-Malpighi Hospital, Via Massarenti 9, 40138 Bologna, Italy; 6 S.C. Fisica Sanitaria, A.O.U. Città Della Salute E Della Scienza Di Torino, Corso Bramante 88, 10126 Torino, Italy; 7 Fisica Sanitaria, ASST Dei Sette Laghi, viale Borri 57, 21020 Varese, Italy; 8 Department of Medical Physics, ASST Grande Ospedale Metropolitano Niguarda, P.za Ospedale Maggiore 3, 20162 Milano, Italy

**Keywords:** Statement, Dose monitoring system, Quality assurance, Radiation dose

## Abstract

The evaluation of radiation burden in vivo is crucial in modern radiology as stated also in the European Directive 2013/59/Euratom—Basic Safety Standard. Although radiation dose monitoring can impact the justification and optimization of radiological procedure, as well as effective patient communication, standardization of radiation monitoring software is far to be achieved. Toward this goal, the Italian Association of Medical Physics (AIFM) published a report describing the state of the art and standard guidelines in radiation dose monitoring system quality assurance. This article reports the AIFM statement about radiation dose monitoring systems (RDMSs) summarizing the different critical points of the systems related to Medical Physicist Expert (MPE) activities before, during, and after their clinical implementation. In particular, the article describes the general aspects of radiation dose data management, radiation dose monitoring systems, data integrity, and data responsibilities. Furthermore, the acceptance tests that need to be implemented and the most relevant dosimetric data for each radiological modalities are reported under the MPE responsibility.

**Supplementary Information:**

The online version contains supplementary material available at 10.1186/s13244-022-01155-1.

## Key points

- Quantitative radiation dose information is essential for justification and optimization in medical imaging.
- Guidelines to assure radiation dose monitoring systems quality and acceptance test.
- Verifying dose data management is crucial before dose monitoring systems clinical implementation.
- MPE is the professional who has important responsibilities for the proper management of dose monitoring.

## Introduction

Optimization and justification of radiological procedures can be effectively achieved only if quantitative information is collected and analyzed in a robust and consistent fashion [1]. In this direction, the implementation of several tools has been proposed over decades by different regulatory and scientific institutions: the International Commission of Radiological Protection published a specific document concerning diagnostic reference levels in radiology as a tool to aid optimization of protection in the medical exposure of patients [2]; the American College of Radiology implemented the Dose Index Registry establishing national benchmarks for CT radiation dose values [3]; and the European Union with the Directive 2013/59/Euratom highlighted the importance of recording, archiving, and communicating dosimetric data [4, 5]. Such requirements can be effectively fulfilled only with the implementation of a computerized tool, namely a radiation dose monitoring system (RDMS; the systems are known also as dose management system [6]), capable of recording and archiving patient data, exposure parameters, as well as radiation dose information.

Despite RDMS increasing use in modern radiology [6–13], currently there are only very few guidelines to assure their quality and their management. In particular, because national and local regulations can require that such systems are medical devices, the need exists to univocally characterize effective quality assurance programs that can guide the MPE and the other radiology professionals to perform acceptance and periodic tests as well as to define the roles and responsibilities of the healthcare professional involved in RDMS management. Furthermore, because the RDMS should record and archive patient data and protected health information, the adequate protection level should be ensured across the whole data management process. In this scenario, the Italian Association of Medical Physics (AIFM—Associazione Italiana di Fisica Medica e Sanitaria) started a specific task group (TG) establishing common guidelines in radiation dose monitoring system management and quality assurance.

Purpose of this manuscript is to report the AIFM statement in radiation dose monitoring systems management and quality assurance. AIFM-TG was established in 2014 and in 2016 published a first report (report n.13) about RDMS management with the contribution of over 70 Medical Physics Expert (MPE) [14]. This manuscript is based on the AIFM-TG work and, after reporting a general RDMS description, focuses on the acceptance tests that the MPE should perform before the clinical implementation of a radiation dose monitoring system. A detailed example of a comprehensive quality assurance report is also provided in the additional files of this article.

## Statement

### Radiation dose monitoring system description and critical points

Current RDMS can either be connected to the radiological devices (rarely) or can be integrated with radiological informatics systems such as radiological information system (RIS) and picture archive and communication system (PACS). When considering data integrity, different data recording methodologies are not equivalent and the data transfer process should be carefully evaluated during acceptance test and before RDMS clinical implementation. In this scenario, the Integrating the Healthcare Enterprise (IHE) initiative recommends to implement the radiation dose structured report (RDSR) as a tool that can comprehensively provide technical parameters, both radiological and geometrical, and dosimetric data in a standardized manner [15]. There are different RDSR templates for different X-rays modalities (CT, radiography, fluoroscopy, and mammography) and for nuclear medicine studies that utilize radiopharmaceuticals. Furthermore, the RDSR is a DICOM object and it can be easily retrieved through the PACS or the RDMS.

Such a scenario is further complicated because, nowadays, all modern radiological devices can provide the RDSR, whereas older devices cannot. Therefore, an efficient radiation dose monitoring system should be able to manage different ways to track and record radiation dose data to accommodate different data formats provided by different modalities [14]. Collecting information included in the image DICOM header allows one to archive exposure and dosimetric data from both old devices and more recent systems with incomplete RDSR data where the implementations do not report all the information needed for patient radiation dose assessment. For instance, the tube current modulation parameters and iterative reconstruction strength are key data in the optimization of CT protocols; however, they are available only in the image DICOM header, but not in the RDSR using the existing DICOM supplement. Moreover, the same issue can be observed in digital radiography exposure index tracking and for some textual descriptive data, such as series descriptions or operator name records [16]. In these contexts, the ideal RDMS should provide a proper combination and an effective visualization of the data simultaneously collected through the RDSR and, in case of missed information, the DICOM header. A last data collection strategy alternative relies on the dosimetric data reported in an image format which can be combined with an OCR text recognition system or with the Modality Performed Procedure Step (MPPS) message. Such an approach certainly provides poorer, less structured, and less robust data; therefore, it should only be implemented when the RDSR is not available or the DICOM header cannot provide suitable information. Care should be exercised in fluoroscopy because fluoroscopic scenes without acquired images are not included in the DICOM images. Lastly, because a high uncertainty is related to manual data entry, it is recommended to implement it only when it is possible to univocally identify the related medical device. In this scenario, it is paramount to effectively train the personnel collecting the data and to estimate the workload related to data integrity and completeness.

In the last decade, several laws and regulations stated the importance of patient radiation dose communication: the European Directive 2013/59/Euratom even required such information to be included in the radiological medical report [4]. Radiation dose communication is not the ultimate RDMS goal; however, it can be achieved through RIS and/or PACS systems that will record the dosimetric data processed and archived in the radiation dose monitoring system. In any case, radiation dose information should not be included in the patient dosimetric record without the final medical physicist approval, particularly image’s exposure index, radiation dose metrics, and organ dose. In this scenario, particular care should be paid to the organs that are not completely included in the exposed area because the related radiation dose could be affected by large errors [7]. Dosimetric data of the population exposed to ionizing radiation for medical purposes, instead, can be sent to local or national public dosimetric registries for statistical and public health analysis. The data must be anonymized and encrypted before the transmission. Furthermore, using this methodology and standardizing the different procedures defined at local and national level using standardized glossaries (RadLex® playbook by the Radiological Society of North America [17, 18]) patient exposures of a specific hospital can be compared with other internationally recognized institutes and reference levels [19].

Radiation dose monitoring systems should be integrated in the radiology department informatics data workflow to manage all the personal and technical data related to the performed procedure. In particular, from a data safety perspective, it is essential to effectively manage the different user credentials.

### Data sources

An important RDMS feature is the ability of acquiring data across all radiology modalities and vendors. Such a requirement is complicated by the differences in dosimetric data format provided by different vendors and different modalities. As briefly introduced in the previous section, all radiological devices should send image data to the PACS. Therefore, a first and highly recommended approach to effective data tracking and monitoring is through a connection of the radiation dose monitoring system with the PACS enabling safe, validated, and consistent data monitoring. Alternatively, it is possible to collect relevant dosimetric, exposure, and patient data building individual connections with each radiological device. Both strategies should rely on a robust quality assurance program to verify and validate the recorded information as described in the following sections.

### Data integrity

According to WHO [20], data integrity (DI) is the degree to which data are complete, consistent, accurate, trustworthy, and reliable. The data and information should also be complete and they should follow the “ALCOA” principles: attributable, legible, contemporaneous, original, and accurate. Verifying the integrity of dosimetric data is part of the radiation dose monitoring system acceptance test [14], with particular regard to data completeness, consistency, and accuracy. Furthermore, data integrity should be ensured also in case of migration to a different RDMS [15].

#### Data completeness

Data completeness in terms of actual availability should be verified considering all the different kinds of data included in the management process. In particular:

- Labels and descriptions (study description, protocol name, anatomical district, projection, etc.);
- Patient data (gender, age, height and weight, etc.);
- Exposure parameters (kV, mA, exposure time, collimation, pitch, scan mode, etc.);
- Radiation dose metrics (KAP, AGD, CTDI, DLP, etc.);
- Derived quantities (SSDE, PSD, Organ dose, effective dose, etc.);

It is paramount to verify the completeness of the data (in terms of nonzero values or empty fields) during acceptance test and also periodically, as individual data may be missing due to equipment software updates or other changes in the architecture of the information system that were not properly communicated to the medical physicist.

#### Data consistency

In any database, it is essential that the data are consistent, namely aligned at all times and updated simultaneously with a single operation. As reported before, a RDMS can track and record data from different modalities and through different connections (RDSR, DICOM header, etc.). Furthermore, the data can be retrieved by applying aggregation functions, namely defining common parameters to query the database. In all these cases, data consistency should be ensured across different systems and modalities. In case of redundancy, the internal consistency of the database between the duplicated information and the derived information must be verified. In particular, consistency across methods used to classify and associate the data to the patient record, together with the description of the study performed (different projections or series for tomographic or fluoroscopic/angiographic procedures), should be verified and univocally coded. A typical issue in data consistency is related to the use of different units, particularly in the description of KAP values. Moreover, it is critical that units displayed and stored at the modality level are consistent with the units archived by the RDMS. If possible, the same unit for each radiation dose indicator should be used within devices of the same modality.

Data consistency may also be affected by the truncation of numerical values with different decimal places, stored in the RDSR, the DICOM Header, or in the RDMS. For instance, the x-ray device current–time product (mAs) can be truncated to the integer value in the DICOM Header resulting in a less accurate information for low mAs procedures such as pediatric examinations.

#### Data accuracy

The accuracy of the data sent to the RDMS and of the data generated by the radiological device should be consistent, provided that the transfer process meets all the necessary data integrity and consistency criteria. For each radiation dose indicator, the accuracy must meet the tolerance defined in the related technical standards and guidelines. Such a tolerance is certified by the manufacturer of the radiological device and it is particularly relevant when comparing data collected across different equipment and facilities. It should be noted that, sometimes, the vendor-specific tolerance for specific setup is 50%: a value that might undermine the RDMS usefulness. Possibly, the improvement of production processes can limit future inaccuracy to 20% or less.

Data accuracy is verified by comparison with the device periodical quality control measurement performed by the medical physicist. The differences between the measured and the indicated data must be in any case lower than that differences reported in the technical standards and in the adopted quality control protocols. Lastly, the evaluation of the measured dosimetric indicators for different exposure parameter combinations can be useful to calculate and integrate in the RDMS a set of corrective factors.

### Data post-processing, derived metric calculations and statistical analysis

In addition to the data obtained from the modalities, the RDMS can provide several derived dosimetric quantities, such as the effective dose, the equivalent dose to organs [6], the size specific dose estimation (SSDE) [21, 22], the entrance dose, and the peak skin dose [23–25]. It can also provide patient anthropometric indicators used in dosimetric assessments such as water equivalent diameter (WED) and patient effective diameter [21, 22]. For all derived quantities that are not imported from the equipment or from the RIS/PACS system, access to the coefficients, reference values, information, and bibliography on the calculation methods used should be guaranteed at the installation and in case of software updates. All the data archived in the RDMS system, including the derived quantities, should be also available in forms of graphs and tables, better if exportable. Lastly, the statistical analysis of the data should enable periodic monitoring of the dosimetric indicators to design and implement optimization actions and to perform comparisons with diagnostic reference levels (DRLs). Before the introduction of RDMS, the comparison with DRLs was usually performed on a sample of data. RDMS, instead, enables such a comparison including all the performed studies. Furthermore, it is possible to establish national and local databases also including stratification for different patient anatomical size and clinical indications [26]. Such systems allow one to also activate alert communication tools that can send a message when an exam results in a radiation dose above a threshold enabling the design and the implementation of the related corrective and preventive actions.

### Roles and responsibilities: the Medical Physicist Expert and other healthcare professionals.

MPE knowledge of the mathematical and physical principles of radiation dosimetry is essential in the definition of effective quality control plans to verify the consistency of the data recorded and stored by radiation dose monitoring systems. The European Directive 2013/59/Euratom states that the MPE is an individual “having the knowledge, training and experience to act or give advice on matters relating to radiation physics applied to medical exposure, whose competence in this respect is recognized by the competent authority” [4]. The same approach is also reported in the REM profile of the IHE Radiology Technical Framework Document: “The Profile focuses on conveying the details of individual irradiation events. A proper radiation exposure management program at an imaging facility would involve a medical physicist and define such things as local policies, local reporting requirements, annual reviews, etc.” [15].

In this scenario, the MPE is responsible for data integrity and storage. The MPE role is also critical when there is the need to correct or modify incorrect dosimetric data due to technical (i.e., calibration) or calculation problems. The tests to verify the integrity of the recorded data, and their frequencies, should be implemented together with system and IT administrators. Data integrity should also be verified during the acceptance test and after any RDMS or radiological device upgrade.

The data provided by the RDMS are a unique source of information that can be used to support effective justification and optimization of the radiological procedures. Such data can be shared, analyzed, and discussed with all healthcare professionals involved in patient care.

### Privacy

Radiation dose monitoring systems also record patient information: data such as name, date of birth, age, and medical records are protected health information. In particular, personal data must be managed according to European Union General Data Protection Regulation (GDPR, in the EU [27]), the Health Insurance Portability and Accountability Act (HIPAA, in the USA [28]), or any other local laws and regulations concerning the protection of patient information. If the monitored data are utilized for research activities, the patient informed consent should be collected or waived by the local authority (ethics committee, internal review board, etc.).

### Dosimetric data communication to patients and healthcare professionals

The communication to the patient of a concise radiological risk index included in the medical report or in a specific dosimetric report is a current and controversial topic. Modality dosimetric index could be used as for instance suggested by the 2020 Italian Consensus Document [29]. Until international consensus documents by scientific societies and national agencies will be published, effective dose could be identified as a metric for this purpose because it is an additional metric enabling the patient dosimetric history description and also the comparison of different radiological procedures. However, effective dose was not defined as a personal risk metric because it is calculated using gender- and age-averaged coefficients and it should not be used to assess personal risk [30–33]. In particular, the recent ICRP publication number 147 [34] recommends that: "Measurable radiation dose quantities should be used where radiation dose information relating to patient exposure forms part of reports for medical radiological procedures, as required by EURATOM 59/2013" (see the paragraph "Recording patient dose information in reports for medical procedures"). In this scenario, it is important to highlight that an effective patient communication should include the discussion of the radiological procedure benefit that should always outweigh the risk.

### Acceptance tests

As reported before, it is the MPE’s responsibility to ensure that a RDMS is suitable for clinical use implementing an effective quality assurance program. In particular, the MPE must perform acceptance tests aiming to evaluate that:

- Input data are present in the database at the requested level of accuracy and consistency (data tracking, consistency and recording) across devices;
- Derived quantities are calculated consistently and in a robust manner, statistical analysis is accurate (data post-processing);
- Output and input data are consistent with each other (data archiving).

To perform such tests, a sample (e.g., 10 entries) of each data per modality should be input in the RDMS verifying the system output at patient, study, and series level. For this purpose, a useful starting point can be the so-called “device form”: a specific form filled for each device and modality connected to the tracking software, where the data completeness is reported (Annex 3–6). Moreover, it is recommended to write a report with the results of each test per each modality including the descriptions of every test that did not pass the acceptance criteria.

A key test, particularly when the data are drawn from different paths (PACS or single device) and different objects (DICOM header, RDSR, or MPPS), is to check that the units and statistic of the recorded data are consistent across quantities (mean, median, maximum, etc.) as well as across devices of the same modality.

### Data tracking

Despite currently there is a great variety among the software commercially available, the radiology community is showing a lack of experience in their choice and their use. In particular, the main requirement for an effective and efficient RDMS is the capability of managing patient archiving, with possible data anonymization, and the capability of managing data from the same patient acquired in different anatomical districts (different acquisition series) and at different times (different accession numbers). Prior to the acceptance and the verification of the installed RDMS, it is important to create a document explicitly describing the system architecture as well as the connection type. Moreover, it should be described if the data populating the database are obtained directly from the equipment, indicating the object or file of origin, or if they are derived, indicating the algorithm that generates the data. Since the system can reach a level of rather complex configurations and can also be updated overtime, the system identification data should be divided in three groups:

- Software data: set of information referring to the RDMS system (Annex 1);
- Equipment data: set of data referring to the modality and the connected equipment (Annex 2);
- Input data from device: set of data transmitted to the RDMS or derived and associated with the modality (Annex 3–6);
- Summary table of the different tests (Annex 7)

For any significant maintenance on the RDMS, on the equipment, or on the RIS-PACS system, the collected data must be re-checked and, if necessary, any version variations of the used software should be recorded.

### Data recording, archiving, and post-processing

As the radiation dose monitoring system may be used to record the patient radiation dose history, before clinical implementation the vendor must provide information about how the software links dosimetric data to the same patient if different exams are performed in different sites or under different patient identifications. For instance, it should be assessed if the software manages centralized registry, according to the PIX profile (Patient Information Cross Reference), defined in IHE IT Infrastructure Technical Framework.

It is paramount to check the proper correspondence dosimetric data in case of the same patient with different accession numbers or identification numbers. Patient ID updated or changed a “merge” style message must be available in RDMS using standard ADT message for example for patient reconciliation. The same test must be completed in such cases when an exam is closed and thereafter opened again to be completed with further diagnostic series. Moreover, if the RDMS performs statistical analysis, it must be checked that the data filtered, elaborated, and extracted are correct. Lastly, which quantities can be statistically analyzed for each modality and if they are customizable, must be known and recorded. Dosimetric reports generated by the software and sent to RIS or PACS must be checked in acceptance and also after each main upgrade of the RDMS software and the RIS-PACS. If possible, the final test report can be digitally signed.

### Radiation dose monitoring system clinical implementation

Different radiological modalities provide different dosimetric data. Nowadays, there is not a standardized approach to the data that needs to be recorded and different RDMS commercial systems, particularly open-source software, follow different monitoring strategies. Because data availability affects the analysis, particularly for organ- or individual-dose estimation, it is useful to define a multi-level approach to the data that a radiation dose monitoring system should record for each radiological modality. In particular, three levels can be identified:

- Level I: Minimum
- Level II: Standard
- Level III: Optimal

The minimum level assures that healthcare professionals can monitor the radiation dose values and can calculate the parameters to verify the compliance with the DRLs. Standard and optimal levels allow accurate dosimetric evaluations to design and implement optimization actions and to calculate organ- and patient-dose metrics.

Table 1 reports the parameters related to the minimum, standard, and optimal monitoring levels. Each level includes the information of the previous one. Such three levels can be implemented in different areas of interest: patient, computed tomography, angiography and cardiac angiography, digital projectional radiography (DR), digital mammography, mobile c-arm fluoroscopy, and cone beam computed tomography.

**Table 1:** Summary of the information recorded by a radiation dose monitoring system per modality and implementation level

	Level	Recorded parameters
Patient information	I	Age, sex
	II	Height, weight
	III	BMI, Patient Effective Diameter
Computed tomography	I	CTDI_vol_ or CTDIw, Phantom Type, DLP, and anatomical district per each series (including localizer, contrast monitoring, etc.)
	II	kV, mAs (minimum and average value), mA (minimum and average value), rotation time, collimation, pitch, slice thickness, and scanning range per each series
	III	Automated tube current modulation system descriptors (Noise Index, Effective mAs, etc.), reconstruction algorithm (if iterative the related strength should also be reported), field of view, if perfusion study the number and acquisition timing should also be reported, “virtual filter” applied to save radiation dose on a particular organ (specify the organ), SSDE, water equivalent diameter, and current profile across z axis
Angiography	I	Total KERMA air product (KAP), fluoroscopy KAP, radiography KAP, anatomical region (i.e., chest, abdomen, etc.)
	II	Total number of exposure events Per each exposure event: kV, mA, mAs, frames/second, filtration, fluoroscopy time, radiography image numbers, KAP and KERMA at the patient entrance reference point, X-ray tube position, source–detector distance (SDD), FOV, radiation field size
	III	Table position, source–skin distance (SSD), indication of the different contribution of air KAP and KERMA per each exposure event and per orientation angle, peak skin dose (PSD)
Digital radiography	I	KAP per each exposure event, anatomical region (i.e., chest, abdomen, etc.)
	II	Per each exposure event: kV, mAs, filtration, source–detector distance (SDD), radiation field size, detector radiation dose, tube orientation, air KERMA at reference point
	III	Source–skin distance (SSD)
Digital mammography	I	Air skin KERMA, AGD per each exposure event
	II	Per each exposure event: kV, mAs, anode, filtration, breast thickness, compression force
	III	Tomosynthesis exposure parameters (number of exposures, angle, kV, mAs, filtration)
Mobile fluoroscopy	I	Total KAP, total fluoroscopy time, and anatomical region (i.e., chest, abdomen, etc.)
	II	Total number of exposure events, fluoroscopy KAP, and radiography KAP Per each exposure event: kV, mA or mAs, frames/second, filtration, source–detector distance (SSD), fluoroscopy time, number of radiographic images, cumulative KERMA at reference point
	III	Source–skin distance (SSD)
Cone beam computed tomography	I, II, III	See “Digital radiography” (typically for dental CBCT) or “Angiography” section. Note that CBCT irradiation events must be clearly labeled as rotational events in the RDSR

## Conclusion

The presented statement can offer unique information to MPE and other healthcare professionals in the implementation and in the quality management of radiation dose monitoring systems. Likewise every other radiological medical device, there is a multitude of vendors and commercial applications that can monitor and record radiation dose information in radiology. In such a heterogeneous scenario, a potential pitfall is the lack of standard procedures and methods in the definition and in the performance of acceptance and periodic quality assurance tests. The AIFM report n.13, summarized in this statement, represents the first attempt in ensuring the effective quality assessment of RDMS.

RDMSs are changing the traditional approach to radiology optimization. Before the implementation of such systems, justification and optimization were based on data collected in small dataset or in phantom-based studies providing information only in a highly constrained setup. However, the implementation of automatic exposure systems, as well as iterative image reconstruction algorithms, changed the traditional relationship between patient size, radiation dose, and radiological device performance [35, 36]. In this scenario, only the evaluation of radiological operations in large patient populations, provided by the RDMS, can enable a patient-centric justification and optimization of the procedure as reported in the EuroSafe recommendation [37].

An open topic in current radiology is the communication to the patient of the radiological study radiation dose as requested also by the new EU Directive 59/2013/Euratom [4]. The AIFM-TG did not provide any recommendation about quantities that should be used to communicate the radiation burden associated with a radiological procedure: standardized guidelines are provided by international regulatory and technical institutions [34]. RDMSs are universally recognized as the primary patient and dosimetric information archiving tool, also for communication purposes. In this scenario, standardization and quality assurance of RDMS are critical to ensure an effective and accurate estimation and communication of radiation dose data.

Newly commercially available radiation dose monitoring software are implementing algorithms, often based on Monte Carlo methods, to calculate more accurate dosimetric metrics (i.e., organ dose), closing the gap between device radiation output and more patient-specific quantities [38, 39]. Future extension of the present statement should also include the evaluation of such new calculation methods as well as their comparison with available gold-standards [38].

## Supplementary Information

**Additional file 1.** **Annex 1.** Software data: set of information referring to the RDMS system. **Annex 2.** Equipment data: set of data referring to the modality and the connected equipment. **Annex 3–6.** Input data from device: set of data transmitted to the RDMS or derived and associated with the modality. **Annex 7.** Summary table of the different tests.

## Data Availability

Full availability.

## Abbreviations

ADT: Admit, discharge and transfer
AGD: Average glandular dose
CBCT: Cone beam computed tomography
CT: Computed tomography
CTDI: Computed tomography dose index
DI: Data integrity
DICOM: Digital imaging and communication in medicine
DLP: Dose-length product
DRL: Diagnostic reference level
FOV: Field of view
IHE: Integrating the Healthcare Enterprise
KAP: Air KERMA area product
MPE: Medical physics expert
MPPS: Modality performed procedure step
OCR: Optical character recognition
PIX: Patient information cross reference
PSD: Peak skin dose
RDMS: Radiation dose monitoring system
RDSR: Radiation dose structured report
RIS: Radiological information system
SDD: Source–detector distance
SSD: Source–skin distance
SSDE: Size-specific dose estimate
WED: Water equivalent diameter
