# Supplementary material for: Statement of the Italian Association of Medical Physics (AIFM) task group on radiation dose monitoring systems
Source: Insights Imaging. 2022 Feb 5;13:23. doi: 10.1186/s13244-022-01155-1 (PMC8818083; doi:10.1186/s13244-022-01155-1)
Supplement: Supplementary file 1 — Additional file 1. Annex 1. Software data: set of information referring to the RDMS system. Annex 2. Equipment data: set of data referring to the modality and the connected equipment. Annex 3–6. Input data from device: set of data transmitted to the RDMS or derived and associated with the modality. Annex 7. Summary table of the different tests. [file 13244_2022_1155_MOESM1_ESM.docx]

**ELECTRONIC SUPPLEMENTARY MATERIAL**

**Annex 1**: example of software report (to be filed before acceptance test).

| Manufacturer |  | |
| --- | --- | --- |
| Software name |  | |
| Software version |  | |
| Installation date |  | |
| Is the software web-based? | □ Yes | □ No |
| Does the system support the integration with RIS/PACS? | □ Yes | □ No |
| System input protocols | □ DICOM  □ IHE  □ HL7 | |
| Data input via | □ Automatic transfer from PACS  □ Automatic transfer from the modality  □ Query/retrieve from RIS/PACS  □ Query/retrieve from the modality | |
| System output protocols | □ DICOM  □ IHE  □ HL7  □ Other | |
| The system can transfer data to | □ PACS  □ RIS  □ RIS and PACS  □ Other__________________ | |
| Is it possible to anonymize patient data? | □ Yes | □ No |
| In case a study was assigned to the wrong patient, is it possible to correctly re-assign it? | □ Yes | □ No |
| Are there statistical analysis tools implemented in the system? | □ Yes | □ No |
| Number of devices per modality | Computed radiography: _________  Digital radiography: ____________  Digital mammography: __________  Computed tomography: _________  Angiography: ________  Fluoroscopy: __________________ | |
| Is it possible to customize the software? | □ Yes | □ No |
|  | Description: | |
| Calculated derived dosimetric quantities? | □ Effective dose  □ Organ dose  □ SSDE  □ Other: ______________ | |
| Is it possible to create different user profiles?  Specify. | □ Yes | □ No |
|  | □ Medical Physicist  □ Radiologist  □ Radiographer  □ Other ______________ | |

**Annex 2**: example of radiological device report (to be filed before the RDMS acceptance test).

| Modality |  |
| --- | --- |
| Manufacturer/ model |  |
| Software name |  |
| Software version |  |
| Installation date |  |
| The device will be connected to the RDMS via | □ Automatic transfer from PACS  □ Automatic transfer from the modality  □ Query/retrieve from RIS/PACS  □ Query/retrieve from the modality |
| Data monitoring level (see Table 1) | □ Level 1 : minimum  □ Level 2: standard  □ Level 3: optimal |
| Data sources | □ DICOM header  □ RDSR  □ MPPS  □ OCR  □ Other ______________ |
| Notes |  |

**Annex 3**: example of RDMS acceptance test report for CT

| Date |  |
| --- | --- |
| Performing Physicist |  |
| Scanner model and vendor |  |
| Location |  |
| Station name |  |
| Software version |  |
| List of most frequently performed exams.  Specify if the device is also used to perform pediatric exams. |  |
| Data sources | □ DICOM header  □ RDSR  □ MPPS  □ OCR  □ Other ______________ |

| **Legend**  For the following sections, in the column “recorded” report:  **✓** if the data is present and correct  **×** if the data is present and non-correct  **NO** is the data is not present  In the “notes” column indicate the unit of the reported physical quantity.  The column lever refers to the section Dose monitoring system clinical implementation of the main document. |
| --- |

1. **Patient information**

Data monitoring level: □ Level 1: minimum □ Level 2: standard □ Level 3: optimal

Specific tests:

| **Information** | **Level** | **Recorded** | **Notes** |
| --- | --- | --- | --- |
| Date of birth (age) | 1 |  |  |
| Sex | 1 |  |  |
| Weight | 2 |  |  |
| Height | 2 |  |  |
| BMI | 3 |  |  |
| Patient Effective Diameter | 3 |  |  |
| Patient Water Equivalent Diameter | 3 |  |  |
| Patient ID | 1 |  |  |

1. **Exam and protocol information**

Data monitoring level: □ Level 1: minimum □ Level 2: standard □ Level 3: optimal

Specific tests:

| **Information** | **Level** | **Recorded** | **Notes** |
| --- | --- | --- | --- |
| Study date and time | 1 |  |  |
| Study ID | 1 |  |  |
| Accession number | 1 |  |  |
| Requested procedure | 1 |  |  |
| Scanning protocol | 1 |  |  |
| Performing radiographer | 3 |  |  |
| Prescriptioner | 3 |  |  |
| Radiologist | 3 |  |  |
| Number of exposure events | 1 |  |  |

1. **Dosimetric and scanning protocol information**

Data monitoring level: □ Level 1: minimum □ Level 2: standard □ Level 3: optimal

Specific tests:

| **Information** | **Level** | **Recorded** | **Note** |  |
| --- | --- | --- | --- | --- |
| Series name | 1 |  |  |  |
| CTDI_vol_ / CTDI_w_ | 1 |  |  |  |
| Phantom type | 1 |  |  |  |
| DLP (mGy⋅cm) | 1 |  |  |  |
| Anatomical district | 1 |  |  |  |
| kVp | 2 |  |  |  |
| Scan type (axial, spiral, etc.) | 1 |  |  |  |
| Rotation time | 2 |  |  |  |
| Tube current (min, max, mean) | 2 |  | Specify how mean current is calculated |  |
| Time-current product (min, max, mean) | 2 |  | Specify how mean value is calculated |  |
| Collimation | 2 |  |  |  |
| Pitch and/or table feed | 2 |  |  |  |
| Reconstructed slice thickness | 2 |  |  |  |
| Scanning length | 2 |  |  |  |
| Automated Tube Current Modulation | 3 |  |  |  |
| Reconstruction algorithm | 3 |  |  |  |
| Iterative reconstruction algorithm | 3 |  |  |  |
| FOV | 3 |  |  |  |
| Virtual filtration | 3 |  | Relevant organs |  |
| Other (specify) |  |  |  |  |
| **Brain perfusion exams** | | | | |
| Number of exposures | 3 |  |  |  |
| Timing | 3 |  |  |  |

1. **Derived metrics**

Data monitoring level: □ Level 1: minimum □ Level 2: standard □ Level 3: optimal

Specific tests:

| **Information** | **Level** | **Recorded** | **Note** |
| --- | --- | --- | --- |
| Effective dose | 3 |  |  |
| SSDE | 3 |  |  |
| Organ dose | 3 |  |  |
| Other (specify) |  |  |  |

**Annex 4**: example of RDMS acceptance test report for angiography

| Date |  |
| --- | --- |
| Performing Physicist |  |
| Scanner model and vendor |  |
| Location |  |
| Station name |  |
| Software version |  |
| List of most frequently performed exams.  Specify if the device is also used to perform pediatric exams. |  |
| Data sources | □ DICOM header  □ RDSR  □ MPPS  □ Other ______________ |

| **Legend**  For the following sections, in the column “recorded” report:  **✓** if the data is present and correct  **×** if the data is present and non-correct  **NO** is the data is not present  In the “notes” column indicate the unit of the reported physical quantity.  The column lever refers to the section Dose monitoring system clinical implementation of the main document. |
| --- |

1. **Patient information**

Data monitoring level: □ Level 1: minimum □ Level 2: standard □ Level 3: optimal

Specific tests:

| **Information** | **Level** | **Recorded** | **Notes** |
| --- | --- | --- | --- |
| Date of birth (age) | 1 |  |  |
| Sex | 1 |  |  |
| Weight | 2 |  |  |
| Height | 2 |  |  |
| BMI | 3 |  |  |
| Patient ID | 1 |  |  |

1. **Exam and protocol information**

Data monitoring level: □ Level 1: minimum □ Level 2: standard □ Level 3: optimal

Specific tests:

| **Information** | **Level** | **Recorded** | **Notes** |
| --- | --- | --- | --- |
| Study date and time | 1 |  |  |
| Study ID | 1 |  |  |
| Accession number | 1 |  |  |
| Requested procedure | 1 |  |  |
| Performed protocol | 1 |  |  |
| Performing radiographer | 3 |  |  |
| Requesting physician | 3 |  |  |
| Performing medical team | 3 |  |  |
| Exposure events | 3 |  |  |

1. **Dosimetric and scanning protocol information**

Data monitoring level: □ Level 1: minimum □ Level 2: standard □ Level 3: optimal

Specific tests:

| **Information** | **Level** | **Recorded** | **Note** |  |
| --- | --- | --- | --- | --- |
| Total KAP | 1 |  |  |  |
| Total fluoroscopy KAP | 1 |  |  |  |
| Total fluorography KAP | 1 |  |  |  |
| ESAK at reference point | 1 |  |  |  |
| Anatomical district | 1 |  |  |  |
| Total number of radiographic images | 2 |  |  |  |
| Total fluoroscopy time | 2 |  |  |  |
| kV | 2 |  |  |  |
| mA or mAs | 2 |  |  |  |
| Frame/s | 2 |  |  |  |
| Added filtration | 2 |  |  |  |
| Source to detector distance | 2 |  |  |  |
| KAP | 2 |  |  |  |
| ESAK at reference point | 2 |  |  |  |
| Tube primary and secondary angle | 2 |  |  |  |
| Field of view | 2 |  |  |  |
| Table height | 3 |  |  |  |
| Source to skin distance | 3 |  |  |  |
| Angular KAP and KERMA distribution | 3 |  |  |  |
| Skin entrance collimation | 3 |  |  |  |
| **Rotational exams** | | | | |
| Describe how the rotational exams are recorded | | | | |

1. **Derived metrics**

Data monitoring level: □ Level 1: minimum □ Level 2: standard □ Level 3: optimal

Specific tests:

| **Information** | **Level** | **Reported** | **Note** |
| --- | --- | --- | --- |
| Skin dose map | 3 |  |  |
| Organ dose | 3 |  |  |
| Effective dose | 3 |  |  |
| Other (specify) |  |  |  |

**Annex 5**: example of RDMS acceptance test report for digital radiography

| Date |  |
| --- | --- |
| Performing Physicist |  |
| Scanner model and vendor |  |
| Location |  |
| Station name |  |
| Software version |  |
| List of most frequently performed exams.  Specify if the device is also used to perform pediatric exams. |  |
| Data sources | □ DICOM header  □ RDSR  □ MPPS  □ Other ______________ |

| **Legend**  For the following sections, in the column “recorded” report:  **✓** if the data is present and correct  **×** if the data is present and non-correct  **NO** is the data is not present  In the “notes” column indicate the unit of the reported physical quantity.  The column lever refers to the section Dose monitoring system clinical implementation of the main document. |
| --- |

1. **Patient information**

Data monitoring level: □ Level 1: minimum □ Level 2: standard □ Level 3: optimal

Specific tests:

| **Information** | **Level** | **Recorded** | **Note** |
| --- | --- | --- | --- |
| Date of birth (age) | 1 |  |  |
| Sex | 1 |  |  |
| Weight | 2 |  |  |
| Height | 2 |  |  |
| BMI | 3 |  |  |
| Patient ID | 1 |  |  |

1. **Exam and protocol information**

Data monitoring level: □ Level 1: minimum □ Level 2: standard □ Level 3: optimal

Specific tests:

| **Information** | **Level** | **Recorded** | **Note** |
| --- | --- | --- | --- |
| Study date and time | 1 |  |  |
| Study ID | 1 |  |  |
| Accession number | 1 |  |  |
| Requested procedure | 1 |  |  |
| Performed protocol | 1 |  |  |
| Performing radiographer | 3 |  |  |
| Requesting physician | 3 |  |  |
| Radiologist | 3 |  |  |
| Total KAP | 1 |  |  |

1. **Dosimetric and scanning protocol information**

Data monitoring level: □ Level 1: minimum □ Level 2: standard □ Level 3: optimal

Specific tests for each irradiation event:

| **Information** | **Level** | **Recorded** | **Note** |
| --- | --- | --- | --- |
| Single exposure KAP | 1 |  |  |
| Anatomical district | 1 |  |  |
| kV | 2 |  |  |
| mA or mAs | 2 |  |  |
| Filtration | 2 |  |  |
| Radiation field size | 2 |  |  |
| Source to detector distance | 2 |  |  |
| Detector dose | 2 |  |  |
| Primary and secondary tube angle | 2 |  |  |
| Entrance skin KERMA | 2 |  |  |
| Table height | 3 |  |  |
| Source to skin distance | 3 |  |  |
| Tomosynthesis information (optional) | 3 |  |  |
| Dual energy information (optional) | 3 |  |  |

1. **Derived metrics**

Data monitoring level: □ Level 1: minimum □ Level 2: standard □ Level 3: optimal

Specific tests:

| **Information** | **Level** | **Recorded** | **Note** |
| --- | --- | --- | --- |
| Organ dose | 3 |  |  |
| Effective dose | 3 |  |  |
| Other (specify) | 3 |  |  |

**Annex 6**: example of RDMS acceptance test report for digital mammography

| Date |  |
| --- | --- |
| Performing Physicist |  |
| Scanner model and vendor |  |
| Location |  |
| Station name |  |
| Software version |  |
| Does the device perform mammography screening exams? | □ Yes □ No |
| Data sources | □ DICOM header  □ RDSR  □ MPPS  □ Other ______________ |

| **Legend**  For the following sections, in the column “recorded” report:  **✓** if the data is present and correct  **×** if the data is present and non-correct  **NO** is the data is not present  In the “notes” column indicate the unit of the reported physical quantity.  The column lever refers to the section Dose monitoring system clinical implementation of the main document. |
| --- |

1. **Patient information**

Data monitoring level: □ Level 1: minimum □ Level 2: standard □ Level 3: optimal

Specific tests:

| **Information** | **Level** | **Recorded** | **Note** |
| --- | --- | --- | --- |
| Date of birth (age) | 1 |  |  |
| Sex | 1 |  |  |
| Patient ID | 1 |  |  |

1. **Exam and protocol information**

Data monitoring level: □ Level 1: minimum □ Level 2: standard □ Level 3: optimal

Specific tests:

| **Information** | **Level** | **Recorded** | **Note** |
| --- | --- | --- | --- |
| Study date and time | 1 |  |  |
| Study ID | 1 |  |  |
| Accession number | 1 |  |  |
| Requested procedure | 1 |  |  |
| Performed protocol | 1 |  |  |
| Performing radiographer | 3 |  |  |
| Requesting physician | 3 |  |  |
| Radiologist | 3 |  |  |

1. **Dosimetric and scanning protocol information**

Data monitoring level: □ Level 1: minimum □ Level 2: standard □ Level 3: optimal

Specific tests for each irradiation event:

| **Information** | **Level** | **Recorded** | **Note** |
| --- | --- | --- | --- |
| Mean glandular dose | 1 |  |  |
| kV | 2 |  |  |
| mAs | 2 |  |  |
| Anode/filtration combination | 2 |  |  |
| Breast thickness | 1 |  |  |
| Compression force | 2 |  |  |
| Tomosynthesis exposure parameters | 3 |  |  |

**Annex 7**: example of End-to-End test checklist

| Date |  |
| --- | --- |
| Performing Physicist |  |
| RDMS name and vendor |  |
| Software version |  |

**End-to-End checklist**

| **Check** | **Test** |
| --- | --- |
| □ | Create a new entry for patient and exam |
| □ | Verify the exam is ported in the radiology department worklist (optional) |
| □ | Perform and close the exam |
| □ | Verify the exam is archived in the RDMS database |
| □ | Create a second exam with a different ID |
| □ | Perform and close the second exam |
| □ | Verify if the second exam is in the RDMS database and correctly assigned to the right patient |
| □ | Verify if the merging exam task has been correctly reported (if available) |
| □ | Delete one exam from the RDMS database |
| □ | Delete one patient from the RDMS database |
| □ | Verify that the images are correctly assigned to a patient (if the function is available) |
